# A Review of Antiplatelet Activity of Traditional Medicinal Herbs on Integrative Medicine Studies

**DOI:** 10.1155/2019/7125162

**Published:** 2019-01-03

**Authors:** Kyungho Kim, Kwang-Il Park

**Affiliations:** Korean Medicine (KM)-Application Center, Korea Institute of Oriental Medicine (KIOM), 70 Cheomdan-ro, Dong-gu, Daegu 41062, Republic of Korea

## Abstract

Thrombotic events mainly occurred by platelet activation and aggregation. The vascular occlusion causes serious disease states such as unstable angina, ischemic stroke, and heart attack. Due to the pervading of thrombotic diseases, new antiplatelet drugs are necessary for preventing and treating arterial thrombosis without adverse side effects. Traditional medicinal herbs have been used for the treatment of human ailments for a long time. The clinically useful and safe products from traditional medicinal herbs were identified and developed in numerous pharmacological approaches. A complementary system of traditional medicinal herbs is a good candidate for pharmacotherapy. However, it still has a limitation in its function and efficacy. Thus, it is necessary to study the mode of action of traditional medicinal herbs as alternative therapeutic agents. In this review, we focused on our current understanding of the regulatory mechanisms of traditional medicinal herbs in antiplatelet activity and antithrombotic effect of traditional medicinal herbs on platelet function.

## 1. Introduction

Thrombosis is one of the leading pathological causes of morbidity and mortality in a wide range of cardiovascular diseases [1]. Thrombus formation is initiated by the adhesion of circulating platelets to the damaged blood vessel walls [2]. Vasoocclusive events are a major cause of death and involve serious vascular diseases such as unstable angina, ischemic stroke, and myocardial infarction [3]. Activation of platelet effector responses (exocytosis and other response independent of exocytosis) triggers the adhesion of platelets to the exposed subendothelial matrix and induces morphological changes, thromboxane A_2_ (TxA_2_) synthesis, and exteriorization of phosphatidylserine [4, 5]. Due to the high prevalence of thrombotic diseases [6], several studies are being carried out on new antithrombotic drugs, which inhibit platelet function, and upstream elements in the signaling cascades that activate platelets [7]. P_2_Y_12_ antagonists are a good example of extensively used in the treatment and prevention of cardiovascular diseases [8]. Although these drugs inhibit the effect of adenosine diphosphate (ADP) and attenuate almost all platelet responses, the predisposing of bleeding is the main off-target effect [9]. Thus, there is a need to develop novel alternative antithrombotic remedies that have limited adverse effects. Traditional medicinal herbs (TMHs) have been considered as an alternative remedy in pharmaceutical industries [10]. Recently, several studies have been demonstrated the antiplatelet, fibrinolytic, and anticoagulant activities of plant extracts or natural products, such as coumarins, xanthones, alkaloids, flavonoids, anthraquinones, naphthalenes, and stilbenes [11–20]. Indeed, the extensive experience with TMHs positions them as good candidates for novel pharmacotherapeutic agents [20, 21]. According to the World Health Organization (WHO) estimates, approximately 80% of the world's population uses TMHs for their primary healthcare [22, 23]. In this review, we focus on the antithrombotic effects of TMHs that regulate platelet activation and aggregation and summarize the mechanisms by which TMHs influence platelet thrombus formation.

## 2. Currently Available Antithrombotic Agents

Three classes of antithrombotic agents, including cyclooxygenase-1 (COX-1) inhibitor (aspirin), adenosine diphosphate (ADP) P_2_Y_12_ receptor antagonists (ticlopidine, clopidogrel, prasugrel, and ticagrelor), and glycoprotein (GP) IIb/IIIa inhibitors (abciximab, eptifibatide, and tirofiban), are currently approved for clinical events in patients undergoing arterial thrombosis [24–27].

### 2.1. COX-1 Inhibitor (Aspirin)

Aspirin is a prototypic antiplatelet agent for treatment of patients with various atherosclerotic diseases [55]. It exerts its effects by inhibiting the activation of COX-1 enzyme which blocks the synthesis of TxA_2_ from arachidonic acid [56]. Aspirin is more effective in the prevention of arterial thrombosis than venous thrombosis [57]. This is attributable to the important role of platelets in the causation of arterial thrombosis. Clinical trials of high-dose aspirin have shown that the antithrombotic efficacy of aspirin can be blunted [58]. Given that thromboxane receptors are expressed in all vascular tissues, including inflammatory cells, endothelial cells, atherosclerotic plaques, and platelets, most of the high doses of aspirin inhibit the activity of COX-1 in all tissues, indicating that the antithrombotic efficacy of high doses of aspirin might have an independent of platelet COX-1 inhibition [59, 60]. Further, numerous studies have shown the risks associated with the use of aspirin for primary prevention of peripheral vascular disease, polycythemia vera, diabetes, end-stage renal disease, and carotid stenosis [61–63]. In addition, long-term aspirin therapy is associated with a modest increase in the incidence of gastrointestinal bleeding [64]. Thus, despite the distinct antithrombotic efficacy of aspirin, its clinical use continues to be suboptimal.

### 2.2. P_2_Y_12_ Receptor Antagonists (Ticlopidine, Clopidogrel, Prasugrel, and Ticagrelor)

Ticlopidine and clopidogrel are prodrugs. These irreversibly bind and inhibit the P_2_Y_12_ receptor for the lifespan of the platelet after* in vivo* bioactivation via the cytochrome P450 (CYP) enzyme system in the liver [65, 66]. Ticlopidine (Ticlid) is an antiplatelet drug in the first thienopyridine that was received by the US Food and Drug Administration (FDA) in 1991 to reduce the incidence of ischemic events in coronary artery disease (CAD) patients. Treatment of ticlopidine (250 mg per twice daily) showed an efficacious antithrombotic effect in patients with peripheral artery bypass surgery, unstable angina, claudication, and cerebrovascular disease [65]. However, in a few cases, treatment of ticlopidine is associated with a high incidence of neutropenia and it is irreversible and potentially fatal [67]. Clopidogrel (Plavix) is an orally available second generation of thienopyridine that was approved by the FDA in 1997 to reduce the ischemic events in patients with atherosclerotic vascular diseases following the results of the CAPRIE (Clopidogrel versus Aspirin in Patients at Risk of Ischemic Events) trial [68]. Although clopidogrel represents an advance in antithrombotic therapy compared with ticlopidine, thrombotic thrombocytopenic purpura (TTP) occurs [69, 70]. Prasugrel (Effient) is a prodrug and orally available third-generation of thienopyridine [71]. Similar to clopidogrel, its active metabolites is regulated by CYP system in the liver and it irreversibly binds to platelet P_2_Y_12_ receptor. However, it is quickly hydrolyzed by intestinal and blood esterases and oxidized more efficiently to its active metabolites through a single CYP-dependent step [71]. In patients with stable coronary disease and elective percutaneous coronary intervention (PCI), prasugrel has a more effective in platelet aggregation compared with clopidogrel [72, 73]. However, its metabolites directly inhibit the function of neutrophils and its use is associated with an increased risk of bleeding [74, 75]. Ticagrelor (Brillinta), also known as AZD6140, is an oral compound belonging to the class of cyclopentyl triazolo-pyrimidine. It is also metabolized via hepatic bioconversion to form an active metabolite [76]. Like the thienopyridines, ticagrelor also directly but reversibly binds to the P_2_Y_12_ receptor on platelet. In this case, the drug displayed only ~30 - 40% of the antiplatelet effect [77, 78]. This may be because ticagrelor interacts with plasma proteins in the circulation [79]. Similar to prasugrel, ticagrelor has been shown to produce a more effective antithrombotic effect compared with clopidogrel in patients irrespectively of genetic differences [80–83]. However, the incidence of dyspnea and hemorrhagic strokes was increased in the ticagrelor-treated group [84, 85]. Given that ticlopidine, clopidogrel, prasugrel, and ticagrelor display a good antithrombotic activity, the treatment of drugs should be defined in the clinical setting and events.

### 2.3. GPIIb/IIIa Inhibitors (Abciximab, Eptifibatide, and Tirofiban)

Abciximab (ReoPro) is the first GPIIb/IIIa antagonist that was approved by the FDA in 1994 for the prevention of ischemic complications of angioplasty [86, 87]. Later, it was approved for PCI with stents and as medical therapy for unstable angina [88, 89]. Most of the administrated abciximab binds to GPIIb/IIIa on platelet with high affinity, but not irreversible, thereby preventing platelet aggregation and thrombus formation. However, long-term treatment of abciximab has shown a quite remarkable mortality in patients with PCI [90, 91]. Abciximab was shown to reduce the risk of death, myocardial infarction, repeat angioplasty, and bypass surgery. However, it may potentially cause fatal bleeding [86, 87]. Eptifibatide (integrilin) is a synthetic cyclic heptapeptide of <1 kDa that was approved by the FDA in 1998. Its design was reliant on a Lys-Gly-Asp (KGD) motif from snake venom disintegrin barbourin that was shown to have potent antiplatelet activity [92]. Eptifibatide acts as a highly potent inhibitor of fibrinogen binding to GPIIb/IIIa and rapidly and reversibly inhibits platelet aggregation with modest prolong bleeding time [93–95]. Eptifibatide has a relatively long plasma half-life but it is primarily removed by kidneys [96]. Although the safety and efficacy of eptifibatide were conducted in different clinical trials, it must be dose reduced in patients with kidney failure and not given to patients receiving dialysis [97–100]. Tirofiban (Aggrastat) is a small molecule based on an RGD-peptidomimetic analog of tyrosine that was approved by the FDA in 1998 [101, 102]. Tirofiban specifically and reversibly binds to GPIIb/IIIa on resting platelets and inhibits the platelet aggregation [101, 102]. The advantages of tirofiban are recovering from platelet aggregation to 50% of the baseline value within 4 hours when an infusion is stopped. Further, it is also removed by kidneys and biliary excretion [103]. Therefore, it is required to dose adjustment of tirofiban in patients with kidney insufficiency. As established by extensive clinical trials and usage of GPIIb/IIIa inhibitors [104], ongoing trials should be required to focus primarily on reduction of side effects including reduction of bleeding and dosage optimization in patients with kidney failure.

## 3. The Benefits of Traditional Medicinal Herbs on Platelet Function

Traditional medicinal herbs (TMHs) are a part of East-Asian medical systems and have been used for the treatment of various diseases [105]. TMHs are now being manufactured as drugs containing ingredients of standardized quality and quantity. Most of the TMHs are relatively low cost, are effective and abundant resources, and have minimized adverse effects in clinical research [106, 107]. Particularly, several studies have demonstrated that most of TMHs showed a positive impact on thrombotic diseases [108]. However, the antithrombotic effect of TMHs on platelet function is relatively unknown. In this review, we will focus on our current understanding of the regulatory mechanisms and the antithrombotic effect of TMHs on platelet function. We judiciously selected total 75 candidates (Table 1) from our database (unpublished data). Among these, only eleven plants have been investigated with respect to their antiplatelet activity, i.e.,* Rhus verniciflua*,* Salvia miltiorrhiza, Caesalpinia Sappan, Curcuma zedoaria*,* Curcuma aromatic, Cinnamomum cassia, Paeonia lactiflora, Panax ginseng, Anemarrhena asphodeloides, Coptis chinensis, *and* Carthamus tinctorius* (Table 2).

### 3.1. Rhus Verniciflua (Toxicodendron vernicifluum)

*Rhus verniciflua*, formerly known as the* Toxicodendron vernicifluum*, is a deciduous tree from Anacardiaceae family, which is widely cultivated in Korea, China, and Japan [109]. Since 15^th^ century AD,* R. verniciflua* has been used as an herbal therapy for the stomach problems, liver detoxification, promoting blood circulation, and removing blood stasis [105, 110, 111]. Although the scientific evidence of* R. verniciflua* is lacking in health remedies,* in vitro* studies, recently, have shown the potential of antithrombotic, antioxidant, antiobesity, anti-inflammatory, antimutagenic, and anticancer activities [28, 29, 111–117]. Particularly, the extracts of* R. verniciflua* exhibit a potent antithrombotic effect in human platelets. A study showed that eight urushiol-type compounds extracted from* R. verniciflua* inhibited ADP- or arachidonic acid- (AA-) induced human platelet aggregation in a dose-dependent manner (IC_50_ value of ~ 5 to 15 *μ*mol/L) [116]. Also, the isomaltol and pentagalloyl glucose from* R. verniciflua* inhibited ADP-, AA-, and collagen-induced human platelet aggregation and relative platelet surface receptors [28]. These results demonstrated that* R. verniciflua* has a potential in antiplatelet activity. Therefore, future study should be suggested to further explore the effects.

### 3.2. Salvia Miltiorrhiza (Asian Red Sage)

*Salvia miltiorrhiza,* also known as Asian red sage, is a medicinal herb for the circulatory system. It is traditionally used for ameliorating the symptoms of cardiovascular and cerebrovascular diseases in Korea, China, and Japan [118–121]. A study has shown that the extract from* S. miltiorrhiza* has beneficial effects on ischemia-induced symptoms including cellular damage and low blood flow [120]. Further, the treatment of* S. miltiorrhiza* in human vein endothelial cells displayed a significant decrease of IL-6 and IL-8, which reflects the effects of* S. miltiorrhiza* on inflammatory responses [122]. The main focus of the predominant bioactivity compounds in* S. miltiorrhiza* is laid on the cardioprotective mechanisms during atherosclerosis, thrombosis, and myocardial infarction by reperfusion [123]. Specifically, the extracts of* S. miltiorrhiza*, including 15, 16-dihydrotanshinone I, lipid-soluble tanshinone I, tanshinone IIA, cryptotanshinone, dihydrotanshinone, water-soluble danshensu, and salvianolic acid B, displayed potent antiplatelet activity via suppression of platelet aggregation and promotion of fibrinolysis [30, 31, 119, 124]. Further, the treatment of* S. miltiorrhiza* successfully prevented blood stasis and ameliorated blood flow from cerebral infarction and hemorrhage [32]. According to clinical studies and the wide range of case studies with* S. miltiorrhiza* after many years of use in Korea, China, and Japan, no major side effects of* S. miltiorrhiza* have been reported, which is extremely safe [31, 125].

### 3.3. Caesalpinia Sappan (Brazilin)

*Caesalpinia sappan*, commonly known as Brazilin or Sappan wood, belongs to the family of Leguminosae. Its dried heartwood has been used as a traditional medicine [126]. Studies have shown that* C. sappan* possesses various pharmacological efficacies such as analgesia, antibacterial, anti-inflammatory antiplatelet activity, promoting blood circulation, and preventing blood stasis [127–130]. The main bioactive component of* C. sappan* is brazilin [7,11b-dihydrobenz(b)indeno[1,2-d]pyran-3,6a,9,10(6H)-tetrol], which has been studied the diverse biological activities such as hypoglycemic, antibacterial, antihepatotoxicity, anti-inflammatory, and anticancer activities [131–134]. A study has shown that brazilin (0.1 to 1 mM) significantly inhibited thrombin-, collagen-, and ADP-induced aggregation of washed rat platelets through a regulation of Ca^2+^ mobilization and phospholipase (PLA_2_) activity [135]. Thus, brazilin may be a useful molecule for the development of a new natural drug for remedying of thrombosis.

### 3.4. Curcuma Zedoaria and Curcuma Aromatic (Turmeric)

*Curcuma zedoaria (*white turmeric) and* Curcuma aromatic* (wild turmeric) are perennial herbs and member of the genus* Curcuma* belonging to the family of Zingiberaceae. These have been used for a traditional medicine in Asia for a long time [33, 136]. Several studies have shown that the drugs of* Curcuma* possess pharmacological effects such as antitumor, anti-inflammatory, antibabesial, immunological activity, cytotoxicity, and antifungal activities [137–142]. Traditionally,* Curcuma* drugs have been used for ameliorating the obstruction of blood circulation. Among them, curcumin (polyphenolic diferuloylmethane) is a major component of* Curcuma *plant [143]. It has a wide range of beneficial effects in cardiovascular disease including antioxidant and anti-inflammatory [144–146]. Intriguingly, curcumin is regarded as a safe compound, because oral administration of curcumin (8g per day) did not show an off-target effect in patients with high-risk or premalignant lesions [147]. Further,* in vitro* studies have shown that curcumin has a significant inhibitory effect in ADP-, AA-, collagen-, platelet activation factor- (PAF-) induced platelet aggregation [34, 35, 148]. Thus, curcumin has a potential in the reduction of platelet aggregation and activation.

### 3.5. Cinnamomum cassia (Cinnamon)

*Cinnamomum cassia*, also known as cinnamon, is an evergreen tree distributed mostly in Asia and member of genus* Cinnamomum* belonging to the family of Lauraceae [36]. The extract of cinnamon is used as a traditional medicine for the alleviation of fever, inflammation, chronic bronchitis, and improving blood circulation [149–151]. The most important constituents of cinnamon are cinnamaldehyde and trans-cinnamaldehyde and other derivatives such as cinnamic acid, coumarins, diterpenoids, and cinnamate [36, 152–154]. These are contributing to the fragrance and various biological activities, including antifungal, antipyretic, antioxidant, and antimicrobial [155–158]. In addition, the extracts of* C. cassia* have found effective inhibition of platelet activation and coagulation [159]. Among the thirteen compounds, eugenol, amygdalactone, cinnamic alcohol, 2-hydroxycinnamaldehyde, 2-methoxycinnamaldehyde, and coniferaldehyde showed a significant inhibitory activity in platelet activation and aggregation compared to acetylsalicylic acid (ASA)[159]. Further, eugenol was previously reported to inhibit platelet activation and aggregation through the suppression of TxA_2_ formation [37, 38]. Thus, the extract of* C. cassia* has a potential for antiplatelet activity.

### 3.6. Paeonia lactiflora (Peony)

*Paeonia lactiflora*, also known as garden peony, is an herbaceous perennial flowering plant in the family of Paeoniaceae and is widespread in Asia [39]. The roots of* P. lactiflora* have long been used under the traditional names of* Paeoniae* Radix in Korea, China, and Japan [160]. It is used as a source of traditional medicine for various diseases such as antipyretic, anti-inflammatory, and analgesic [40, 42, 161]. Particularly, the extract of* Paeoniae* Radix has been used as remedies for cardiovascular diseases for improving blood circulation [162, 163]. Biochemical studies showed that paeonol, a representative component of* Paeonia*, inhibited ADP-, AA-, and collagen-induced platelet aggregation via the inhibition of TxA_2_ and PGD_2_ formation [42, 164]. Further, the extract of* Paeoniae* Radix, including paeoniflorin, benzoylpaeoniflorin, benzoyloxypaeoniflorin, methyl gallate, catechin, paeoniflorigenone, galloylpaeoniflorin, and daucosterol, showed an improving blood circulation through their inhibitory effects on both platelet aggregation and blood coagulation [160]. However, the role of each constituent and their overall effects* in vivo* still remain elusive.

### 3.7. Panax Ginseng (Ginseng)

Ginseng is the root of plants in the genus* Panax*, which includes several species such as Korean ginseng (*Panax ginseng*), South China ginseng (*Panax notoginseng*), and American ginseng (*Panax quinquefolius*) [41]. Ginseng is regarded as a valuable traditional medicine for treatment of different ailments and enhancing immunity. Although ginseng acts as a panacea and heals all kinds of illnesses for a long time, there is little evidence from clinical research [41, 107]. Recently, several studies have focused on the effects of ginseng in vasorelaxant, antioxidant, anti-inflammatory, and antiplatelet properties [41, 165–168]. Particularly, the oral administration of* P. *ginseng extract (daily at doses of 250 and 500 mg/kg) significantly inhibited ADP- and collagen-induced aggregation and granules secretion in rat platelets [169]. Also, the extract of* P.* notoginseng inhibited collagen-induced platelet aggregation by 60% at 3 mg/ml [168]. Biochemical studies showed that ginseng contains various active constituents including ginsenosides, peptides, polysaccharides, mineral oils, and fatty acids [170]. Among them, single ginsenosides, such as Rg1, Rg3, and Rp4, showed a significant reduction of platelet aggregation and Ca^2+^ mobilization via the regulation of PI3K/Akt signaling pathway [44, 45, 171]. Thus, the constituents of ginseng are important for regulating platelet activation and aggregation.

### 3.8. Anemarrhena asphodeloides (Liliaceae)

*Anemarrhena asphodeloides* is an herbaceous plant and member of genus* Anemarrhena *belonging to Asparagaceae family and mainly distributed in Korea, China, and Mongolia [43]. It has been commonly used in traditional medicine for thousands of years [108]. The curative properties of* A. asphodeloides *have been known to have an antidiabetic, antiplatelet, and diuretic activities [172–174]. Further, biochemical studies have shown that the extract of A.* asphodeloides* displayed beneficial effects on the central nervous system, gastric cancer, and inflammation [43, 108, 175]. The primarily compounds isolated from* A. asphodeloides* are xanthones, steroidal saponins, flavonoids, norlignans, and polysaccharides [43, 46, 172, 176]. Particularly, the series of steroidal saponins, including timosaponin A-III, timosaponin B-II, and anemarsaponin B, remarkably inhibited ADP-induced platelet aggregation and delayed thromboplastin times [47, 48, 176, 177]. These results suggested that the steroidal saponins isolated from* A. asphodeloides* might be used as a novel antithrombotic therapeutic agent.

### 3.9. Coptis chinensis (Goldthread)

*Coptis chinensis* is a low-growing plant belonging to Ranunculaceae family. It is indigenous to the mountainous regions of Korea, China, and Japan [49]. The rhizome of* C. chinensis* has been widely used as a tonic remedy for hepatic and cardiovascular disorders for a long time in traditional medicine [178]. Further, pharmacological studies have shown that* C. chinensis* possesses a wide range of beneficial effects in bacterial infection, cancer, and inflammation [179–181]. According to biochemical studies, berberine (5,6-dihydro-9,10-dimethoxybenzo[g]-1,3-benzodioxolo[5,6-a]quinolizinium, BBR) is the major constituent of* C. chinensis *[182]. The beneficial effects of BBR have been reported in carbohydrate and lipid metabolism, inflammation, endothelial function, and cardiovascular system [49, 183–187]. BBR also has an antiplatelet effect that is mediated via the inhibition of arachidonic acid (AA) metabolism and Ca^2+^ mobilization [188]. A study examined that BBR (0.5 mol/L) inhibited collagen-, ADP-, and AA-induced TxA_2_ synthesis in rabbit platelets [188]. Intriguingly, BBR directly interacted with thrombin (Kd value of 16.39 *μ*M), thereby inhibiting thrombin-induced platelet aggregation [50]. Thus, BBR may be a considerable and potential candidate for the development of safe and effective antiplatelet agents.

### 3.10. Carthamus tinctorius (Safflower)

*Carthamus tinctorius*, commonly known as safflower, is an herbaceous and thistle-like annual plant and belongs to the family of Compositae [51]. Its extract and oil are important for use in traditional medicines as a purgative, analgesic, antipyretic, and antidote to poisoning [51, 189]. In Korea,* C. tinctorius* is also known as Honghwain, and it has been clinically used to promote bone formation and prevent menstrual problems, postpartum hemorrhage, and osteoporosis [190, 191]. Further, several clinical studies have investigated the mechanisms of the therapeutic effect of* C. tinctorius* against a diverse range of diseases [192]. The extract of* C. tinctorius* was shown to inhibit platelet aggregation induced by ADP and platelet activating factor (PAF) stimulation, both* in vitro* and* in vivo *[52, 193]. The aqueous extract of* C. tinctorius* also displayed antithrombotic activity against venous thrombosis* in vivo *[53]. Further, the extracts of* C. tinctorius* prolonged the plasma thrombin time (TT), prothrombin time (PT), and activated partial thromboplastin time (APTT) [52, 193]. Thus, the constituents of* C. tinctorius* are important for regulating thrombosis.

## 4. The Prescriptions of Korean Medicine on Platelet Function

Most Asian countries have their own traditional medicines and prescriptions for a long time. Korean traditional medicines (KTM) are widely used for the treatment of various diseases in clinics in Korea [54]. Due to the geomorphological characteristics, Korea has a plenty of herbal plant materials including about 3,400 species, 762 varieties, and 287 forms. Among them, 300 kinds of natural plants are currently used as traditional medicines [194]. Since 1991, the Korean government has attempted to establish the standard of KTM preparations such as manufacturing process, quality control, and handling of KTM [194]. The establishment of the Korean government policy framework and the efforts of the Korea Institute of Oriental Medicine (KIOM) institution have helped standardize the manufacture of KTM preparations using pharmaceutical approaches. In addition, the optimal prescriptions of KTM are studied based on the philosophy of ancient medical science and originated from eleven oriental books in Korea [194]. Among these, we have found the twenty-six prescriptions of KTM based on the Dong-Eu-Bo-Gam (by Hur Joon, AD1713), Je-Jung-Sin-Pyeon (by Kang Myeonggil, AD1799), Gyeong-Ag-Jeon-Seo (by Jang Gaebin, BC 1624), Hwa-Je-Gug-Bang (by Jin samun AD1078), Geum-Gwe-Yo-Lag (by Jang Jungkyung, BC 250), and Ui-Lim-Gae-Chag (by Wang cheongim BC1830), which had a significant efficacy of blood circulation and stasis (Table 3). Further, we found that eight prescriptions of KTM (unpublished data), including Do-Haeg-Seung-Gi-Tang, Bo-Yang-Hwan-O-Tang, On-Gyeong-Tang, Byeol-Gab-Jeon-Hwan, Tong-Gyu-Hwal-Hyeol-Tang, Tal-Hwa-Jeon, So-Pung-Hwal-Hyeol-Tang, and Saeng-Hwa-Tang, had a significant inhibitory effect on platelet aggregation following collagen stimulation (Table 4). These findings might provide the standardization, regulation, and quality control of KTM in the future antithrombotic studies.

## 5. Concluding Remarks

The pathophysiological role of platelet during vascular disease has long been considered to be important. Platelet aggregation and activation are a major cause of cardiovascular disease. Because of the side effects of current antiplatelet agents, TMHs have been mentioned as alternative therapeutic agents. TMHs have been traditionally used in the management of cardiovascular diseases and its progression, particularly, in thrombosis and coagulation. In this review, we give a brief overview of some current platelet receptor antagonists and their main disadvantages. Further, we focused on the bioavailability of TMHs that possess antithrombotic properties. However, only preliminary evidence of the usefulness of TMHs is currently available. Therefore, further studies are required to assess the bioavailability of TMHs and to compare their therapeutic efficacy against the currently FDA-approved platelet receptor antagonists. A better understanding of the mechanisms mediating the bioavailability of TMHs could lead to the identification of a novel therapeutic target for the prevention and treatment of thrombotic diseases.

## Figures and Tables

**Table 1:** The list of traditional medicinal herbs (TMHs).

**Species**	**Vernacular name**	**Species**	**Vernacular name**
*Glycyrrhiza uralensis*	Kamcho	*Trogopterus xanthipes*	Oryunggi
*Curcuma aromatica*	Kanghwang	*Vaccaria segetalis*	Wangbuluhaeng
*Rhus verniciflua*	Kunchil	*Achyranthes bidentate*	Usul
*Spatholobus suberectus*	Kaehyuldeung	*Curcuma aromatica*	Ulgum
*Sophora flavescens*	Kosam	*Artemisia anomala*	Uukino
*Eriocaulon sieboldianum*	Kokjungcho	*Cinnamomum cassia*	Yukkye
*Sophora japonica*	Keuigak	*Leonurus sibiricus*	Ickmocho
*Selaginella tamariscina*	Kwonbaek	*Paeonia lactiflora*	Chokchayak
*Lonicera japonica*	Kumunhwa	*Lyceum chinense*	Ghigolpi
*Phragmites communis*	Nogun	*Viola yedoensis*	Chahwagijeong
*Phaseolus radiates*	Nokdu	*Lithospermum erythrorhizon*	Chacho
*Rhaponticum uniflorum*	Nuro	*Eupolyphaga sinensis*	Chachung
*Salvia miltiorrhiza*	Dansam	*Panax notoginseng*	Cheonchil
*Lophatherum gracile*	Damchukyup	*Citrus unshiu*	Chinpi
*Isatis indigotica*	Daechongyup	*Gleditsia sinensis*	Chogakcha
*Glycine max*	Daeduhwangkwon	*Anemarrhena asphodeloides*	Chimo
*Rheum palmatum*	Daehwang	*Phyllostachys nigra, henonis*	Chukyup
*Prunus persica*	Doin	*Cnidium officinale*	Chunking
*Lasiosphaera fenzlii*	Mabal	*Manis pentadactyla*	Chunsangap
*Portulaca oleracea*	Machihyun	*Artemisia annua*	Chungho
*Verbena officinalis*	Mapyuncho	*Gentian scabra*	Choyoungdam
*Tabanus bivittatus*	Mangchung	*Leonurus sibiricus*	Chungulcha
*Erigeron canadensis*	Mangcho	*Patrinia villosa*	Paechangkun
*Paeonia suffruticosa*	Mokdanpi	*Taraxacum mongolicum*	Pogongyoung
*Buddleja officinalis*	Milmonghwa	*Smilax glabra*	Tobokrung
*Pulsatilla koreana*	Baekduong	*Lycopus lucidus*	Taklan
*Dictamnus dasycarpus*	Baeksunpi	*Scrophularia buergeriana*	Hyunsam
*Oldenlandia diffusa*	Baekwhasasulcho	*Corydalis turtschaninovii*	Hyunhosak
*Belamcanda chinensis*	Sakan	*Coptis chinensis*	Hwangnyon
*Sophora tonkinensis*	Sandukeon	*Carthamus tinctorius*	Honghwa
*Cremastra appendiculata*	Sanchako	*Polygonum cuspidatum *	Hojangkun
*Sparganium stoloniferum*	Samneong	*Prunella vulgaris lilacina*	Hagocho
*Whitmania pigra*	Suchil	*Scutellaria baicalensis*	Hwangkun
*Rehmannia glutinosa*	Sukchihwang	*Caesalpinia sappan*	Somok
*Massa Medicate Fermentat*	Singok	*Nelumbo nucifera*	Yeonchayuk
*Curcuma zedoaria*	Achul	*Lonicera japonica*	Indong
*Chrysanthemum indicum*	Yaguk	*Stellaria dichotoma*	Ensiho
*Houttuynia cordata*	Erseoungcho		

**Table 2:** The active constituents of TMHs.

**Scientific name**	**Active compound**	**Structure**	**Results**	**Ref.**
*Rhus verniciflua*	3-(8′R,9′R-dihydorxypentadecyl)-phenol	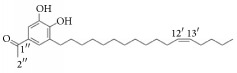	Inhibition of platelet aggregation induced by ADP and AA.	[28]
	1-[3,4-dihydroxy-5-(12′Z)-12-heptadecen-1-ylphenyl]-ethanone	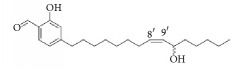		
	Isomaltol	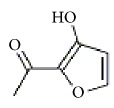	Inhibition of platelet aggregation induced by ADP, AA, collagen.	[29]
	Pentagalloyl glucose	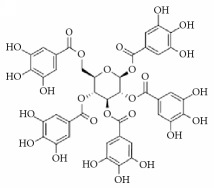		

*Salvia miltiorrhiza*	15,16-dihydrotanshinone I	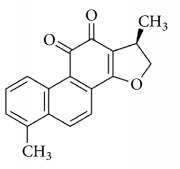	Inhibited collagen-induced platelet aggregation via Ca^2+^ mobilization and TxA_2_ generation, Inhibited AA metabolism.	[30–32]
	Tanshinone I	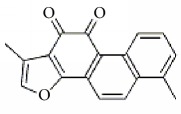		
	Tanshinone IIA	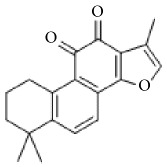		
	Cryptotanshinone	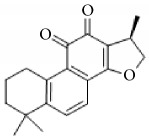		
	Danshensu	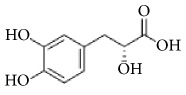		
	Salvianolic acid B	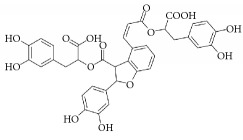		

Caesalpinia sappan	Brazilin	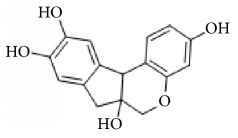	Inhibited platelet aggregation activity induced by thrombin, collagen, and ADP.	[33]

*Curcuma zedoaria*	Curcumin	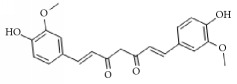	Inhibitory effect in ADP-, AA-, collagen-, platelet activation factor (PAF)-induced platelet aggregation.	[34–36]
*Curcuma aromatic*				

*Cinnamomum cassia*	Eugenol	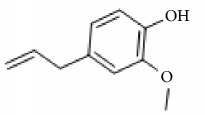	Inhibition of ADP-, collagen-, AA-induced platelet activation and aggregation. Inhibitory effect in TxA_2_ formation and Ca2^+^ mobilization.	[37–39]
	Amygdalactone	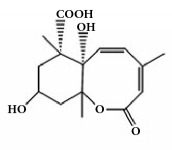		
	Cinnamic alcohol	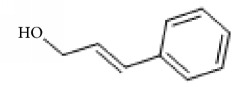		
	2-Hydroxycinnamaldehyde	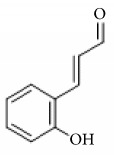		
	2-Methoxycinnamaldehyde	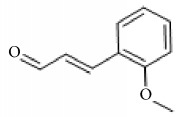		
	Coniferaldehyde	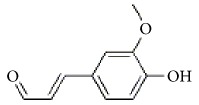		

*Paeonia lactiflora*	Paeonol	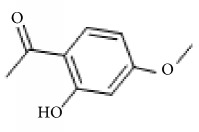	Inhibited ADP-, AA-, and collagen-induced platelet aggregation via the inhibition of TxA_2_ and PGD_2_ formation.	[40, 41]
	Paeoniflorin	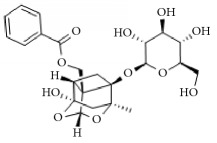	Improving blood circulation through anti-platelet aggregation and blood coagulation.	[42]
	Benzoylpaeoniflorin	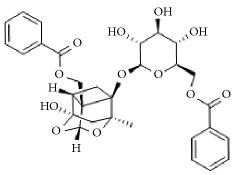		
	Benzoyloxypaeoniflorin	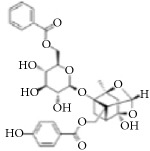		
	Methyl gallate	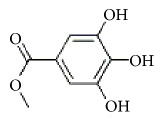		
	Catechin	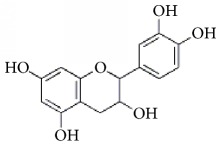		
	Paeoniflorigenone	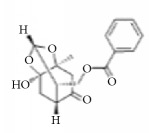		
	Galloylpaeoniflorin	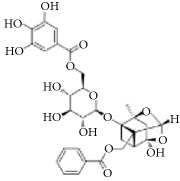		
	Daucosterol	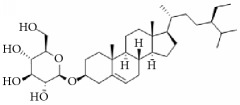		

*Panax ginseng*	Ginsenoside Rg1	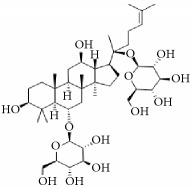	Inhibition of platelet activation and aggregation induced by thrombin, ADP, collagen, and U46619.	[43]
	Ginsenoside Rg3	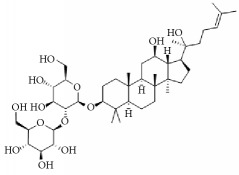		[44]
	Ginsenoside Rp4	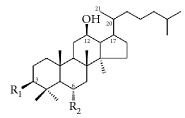		[45]

*Anemarrhena asphodeloides*	Timosaponin A-III	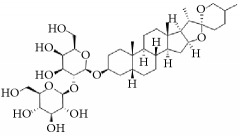	Remarkably inhibited ADP-induced platelet aggregation and delayed thromboplastin time.	[46–49]
	Timosaponin B-II	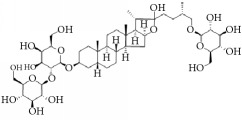		
	Anemarsaponin B	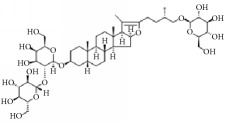		

*Coptis chinensis*	Berberine	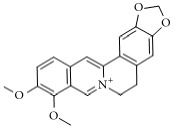	Inhibited ADP, collagen, AA-induced platelet aggregation and TxA_2_ synthesis.	[50, 51]

*Carthamus tinctorius*	Hydroxysafflor yellow A	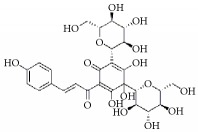	Inhibited ADP, PAF induced platelet aggregation and delated PT, TT and APTT	[52–54]

**Table 3:** The prescriptions of Korean traditional medicine.

**Prescription**	**Components**	**Literatures**
On-Gyeong-Tang	Big blue lilyturf (8g), Korean Angelica (6g), Ginseng (4g), Pinellia (4g), white peony (4g), Cnidium (4g), Moutan (4g), Gelatinum (3g), licorice (3g), Evodia (2g), Cinnamon (2g)	Dong-Eu-Bo-Gam

Cheon-Geum-Jo-Gyeong-Tang	Big blue lilyturf (8g), Korean Angelica (6g), Ginseng (4g), Pinellia (4g), white peony (4g), Cnidium (4g), Moutan (4g), Gelatinum (2g), Evodia (2g), Cinnamon (2g), Ginger (2g)	Dong-Eu-Bo-Gam

Dae-Hwang-Mog-Dan-PI-Tang I	Moutan (10g), licorice (6g), Rhubarb (6g), Peach kernel (10g), Kirilowii (10g)	Dong-Eu-Bo-Gam

Dae-Hwang-Mog-Dan-PI-Tang II	Moutan (10g), licorice (6g), Rhubarb (6g), Peach kernel (10g), Gourd (10g)	Dong-Eu-Bo-Gam

Bog-Won-Hwal-Hyeol-Tang	Korean angelica (6.8g), licorice (4g), Rhubarb (10g), Peach kernel (10g), Bupleurum (6g), Pangolin (4g), Dogeun (4g), Safflower (2g)	Dong-Eu-Bo-Gam

Byeol-Gab-Jeon-Hwan	Peach kernel (20g), Safflower (20g), Amyda shell (40g), Bur-reed (20g), Curcuma (20g), Cyperus (20g), Nastsudaidai peal (20g), Nuruk (20g), Malt (20g), Shell powder (20g)	Dong-Eu-Bo-Gam

Gwi-Chul-Pa-Jing-Tang	white peony (3.75g), Safflower (1.87g), Bur-reed (3.75g), Curcuma (3.75g), Cyperus (5.62g), Nastsudaidai peal (3.75g), Peony (3.75g), Dong quai (3.75g), Lindera root (2.6g), Sappan (1.87g), Cinnamon (1.87g)	Je-Jung-Sin-Pyeon

Do-Haeg-Seung-Gi-Tang	licorice (3.7g), Cinnamon (7.5g), Rhubarb (11.2g), Peach kernel (10g), Glauber salt (7.5g)	Je-Jung-Sin-Pyeon

Tong-Gyeong-Tang	Korean angelica (2.6g), white peony (2.6g), Rhubarb (2.6g), Safflower (2.6g), Sappan (2.6g), Cinnamon (2.6g), Rehmania (2.6g), Machilia (2.6g), Citrus (2.6g), Poncirus (2.6g), Orpiment (2.6g), Mume fruit (2g), Ginger (3g), Jujube (2g)	Je-Jung-Sin-Pyeon

Tong-Gyeong-Tang + Hwanglyeon	Korean angelica (2.6g), white peony (2.6g), Rhubarb (2.6g), Safflower (2.6g), Sappan (2.6g), Cinnamon (2.6g), Rehmania (2.6g), Machilia (2.6g), Citrus (2.6g), Poncirus (2.6g), Orpiment (2.6g), Mume fruit (2g), Ginger (3g), Jujube (2g), Coptis (2.6g)	Je-Jung-Sin-Pyeon

Hwal-Hyeol-Tang	Cnidium (2.6g), Moutan (3.7g), licorice (0.75g), Cinnamon (1.87g), Ginger (1g), Peach kernel (3.7g), Safflower (2.6g), Cyperus (3.7g), Peony (3.75g), Dong Quai (3.75g), Lindera root (3.75g), Citrus (3.75g), Corydalis (3.75g), Elecampane (1.87g)	Je-Jung-Sin-Pyeon

Tong-Gyu-Hwal-Hyeol-Tang	Korean Angelica (6g), Ginseng (2g), licorice (2g), Bupleurum (4g), Nastsudaidai peal (1.2g), Milk Vetch root (4g), Atractylodes (4g), Cimicifuga (4g), Anemarrhena (4g), Ostericum (4g), Seseleos radix (2g), Angelica dahurica (2g), Orpiment (2g), Alisma (2g), Orange peel (1.2g), Coptis (1.2g), Elecampane (1.2g)	Dong-Eu-Bo-Gam

Bo-Yang-Hwan-O-Tang,	Korean Angelica (1.2g), Ginseng (2g), licorice (2g), Bupleurum (6g), Peony (1.2g), Rehmannia (2g), Milk Vetch root (2g), Atractylodes (2g), Anemarrhena (1.2g), Ostericum (2g), Seseleos radix (1.2g), Alisma (1.2g), Orange peel (1.2g), Crudes (1.2g), White Poria cocos (1.2g), Cinnamon(1.2g)	Dong-Eu-Bo-Gam

Sil-So-San	*Trogopterorum faeces* (4g), *Typhae Pollen* (4g)	Dong-Eu-Bo-Gam

Gye-Ji-Bog-Lyeong-Hwan	Moutan (4g), Peach kernel (4g), Peony (4g),. Red Poria cocos (4g), Cinnamon (4g)	Dong-Eu-Bo-Gam

Gyeong-Ha-Chul-Eo-Tang	Cnidium (7.5g) Moutan (7.5g), licorice (3.7g), Peach kernel (2.6g), Safflower (2.6g), Cyperus (2.6g), Peony (2.6g), Korean Angelica (2.6g), Lindera root (2.6g), Citrus (7.5g), Corydalis (3.75g), *Trogopterorum faeces* (3.75g)	Ui-Lim-Gae-Chag

Dae-Hwang-Ja-Chung-Hwan	licorice (75g), Rhubarb (37.5g), Safflower (3.75g) Peony (112.5g), Rehmania (375g), Orpiment (75g), Eupolyphaga (37.5g), Lacquer tree bark (37.5g), Hirudo (37.5g), Breeze (37.5g), Styrax (37.5g), *Persicae Semen* (243.75g)	Geum-Gwe-Yo-Lag

Tal-Hwa-Jeon	Korean Angelica (26.2g), Cinnamon (3.75g), *Cnidii Rhizoma* (7.5g), Achyranthes (7.5g), Psyllium (5.62g)	Dong-Eu-Bo-Gam

So-Bog-Chug-Eo-Tang	Korean Angelica (11.2g), Cnidium (7.5g), Cinnamon (3.75g), Ginger (5g), Peony (7.5g), Thphae Pollen (11.2g),* Trogopterorum faeces* (7.5g), Myrrha (7.5g), *Foeniculi Fructus* (7.5g), Corydaline (3.75g),* Zingiberis Rhizoma *(3.75g)	Je-Jung-Sin-Pyeon

So-Pung-Hwal-Hyeol-Tang	Korean Angelica (3.7g), Cnidium (7.5g), Safflower (1.12g), Atractylodes (3.75g), *Angelica dahurica*(2g), Orpiment 3.75g), *Clematidis Radix* 3.75g), *Stephaniae TetrandraeRadix* (3.75g), *ArisamatisRhizoma* (3.75g), *OstericiiRadix *(3.75g), *Cinnamomi Ramulus* (3.75g)	Je-Jung-Sin-Pyeon

Hwal-Lag-Hyo-Lyeong-Dan	Korean Angelica (18.7g), Myrrha (18.7g), *Salvia miltiorrhiza* (18.7g), Frankincense (18.7g)	Je-Jung-Sin-Pyeon

So-Hwal-Lag-Dan	Myrrha (7.5g), *Aconiti Radix* (22.5g), *Aconiti Ciliare Tuber* (22.5g), *Arisamatis Rhizoma *(22.5g), Lumbricus (22.5g), Olibanum (7.5g)	Hwa-Je-Gug-Bang

Saeng-Hwa-Tang	Korean angelica (18.7g), Cnidium (7.5g), Peach kernel (10g), Rehmania (11.25g), Jujube (2g), licorice (1.87g), *Zingiberis Rhizoma* (1.12g)	Gyeong-Ag-Jeon-Seo

**Table 4:** The antiplatelet activity of Korean medicine prescriptions.

**Sample**	**The inhibition of rat platelet aggregation following collagen stimulation**
Do-Haeg-Seung-Gi-Tang	65.9 ± 3.8

Bo-Yang-Hwan-O-Tang	54.6 ± 6.43

On-Gyeong-Tang	4.7 ± 0.2

Byeol-Gab-Jeon-Hwan	1.5 ± 6.9

Tong-Gyu-Hwal-Hyeol-Tang	13.6 ± 6.43

Tal-Hwa-Jeon	8.9 ± 6.5

So-Pung-Hwal-Hyeol-Tang	13.6

Saeng-Hwa-Tang	17.1 ± 10.8

All samples were prepared as described in Table 3. Washed platelets in HEPES-Tyrode buffer were preincubated with 0.01% DMSO or 100 *μ*g/ml of samples for 10 minutes at 37°C and then stimulated with collagen (1 *μ*g/ml). Platelet aggregation was monitored in a platelet aggregometer (Chronolog Corp., Havertown, PA) at 37°C with stirring (1,000 rpm).

## References

[1] Benjamin E. J., Virani S. S., Callaway C. W. (2018). Heart Disease and Stroke Statistics-2018 Update: A Report From the American Heart Association. *Circulation*.

[2] Furie B., Furie B. C. (2008). Mechanisms of thrombus formation. *The New England Journal of Medicine*.

[3] Libby P., Ridker P. M., Maseri A. (2002). Inflammation and atherosclerosis. *Circulation*.

[4] Reed G. L., Fitzgerald M. L., Polgar J. (2000). Molecular mechanisms of platelet exocytosis: insights into the ‘secrete’ life of thrombocytes. *Blood*.

[5] Golebiewska E. M., Poole A. W. (2015). Platelet secretion: From haemostasis to wound healing and beyond. *Blood Reviews*.

[6] Raskob G. E., Angchaisuksiri P., Blanco A. N. (2014). Thrombosis: A Major Contributor to Global Disease Burden. *Seminars in Thrombosis and Hemostasis*.

[7] Capodanno D., Ferreiro J. L., Angiolillo D. J. (2013). Antiplatelet therapy: New pharmacological agents and changing paradigms. *Journal of Thrombosis and Haemostasis*.

[8] Meadows T. A., Bhatt D. L. (2007). Clinical aspects of platelet inhibitors and thrombus formation. *Circulation Research*.

[9] Becker R. C., Bassand J. P., Budaj A. (2011). Bleeding complications with the P2Y12 receptor antagonists clopidogrel and ticagrelor in the PLATelet inhibition and patient Outcomes (PLATO) trial. *Eur Heart J*.

[10] Tognolini M., Barocelli E., Ballabeni V. (2006). Comparative screening of plant essential oils: phenylpropanoid moiety as basic core for antiplatelet activity. *Life Sciences*.

[11] Saluk-Juszczak J., Pawlaczyk I., Olas B. (2010). The effect of polyphenolic-polysaccharide conjugates from selected medicinal plants of Asteraceae family on the peroxynitrite-induced changes in blood platelet proteins. *International Journal of Biological Macromolecules*.

[12] Mekhfi H., Haouari M. E., Legssyer A. (2004). Platelet anti-aggregant property of some Moroccan medicinal plants. *Journal of Ethnopharmacology*.

[13] Kontogiorgis C., Nicolotti O., Mangiatordi G. F. (2015). Studies on the antiplatelet and antithrombotic profile of anti-inflammatory coumarin derivatives. *Journal of Enzyme Inhibition and Medicinal Chemistry*.

[14] Lee W., Lee J., Kulkarni R. (2016). Antithrombotic and antiplatelet activities of small-molecule alkaloids from Scolopendra subspinipes mutilans. *Scientific Reports*.

[15] Seo E. J., Ngoc T. M., Lee S.-M., Kim Y. S., Jung Y.-S. (2012). Chrysophanol-8-*O*-glucoside, an anthraquinone derivative in rhubarb, has antiplatelet and anticoagulant activities. *Journal of Pharmacological Sciences*.

[16] Yoo H., Ku S.-K., Lee W. (2014). Antiplatelet, anticoagulant, and profibrinolytic activities of cudratricusxanthone A. *Archives of Pharmacal Research*.

[17] Giordanetto F., Wallberg A., Ghosal S. (2012). Discovery of phosphoinositide 3-kinases (PI3K) p110beta isoform inhibitor 4-[2-hydroxyethyl(1-naphthylmethyl)amino]-6-[(2S)-2-methylmorpholin-4-yl]-1H-pyri midin-2-one, an effective antithrombotic agent without associated bleeding and insulin resistance. *Bioorganic & Medicinal Chemistry Letters*.

[18] Yang N.-Y., Tao W.-W., Duan J.-A. (2010). Antithrombotic flavonoids from the faeces of Trogopterus xanthipes. *Natural Product Research*.

[19] Messina F., Guglielmini G., Curini M., Orsini S., Gresele P., Marcotullio M. C. (2015). Effect of substituted stilbenes on platelet function. *Fitoterapia*.

[20] Moeini R., Memariani Z., Pasalar P., Gorji N. (2017). Historical root of precision medicine: an ancient concept concordant with the modern pharmacotherapy. *DARU Journal of Pharmaceutical Sciences*.

[21] Memariani Z., Moeini R., Hamedi S. S., Gorji N., Mozaffarpur S. A. (2018). Medicinal plants with antithrombotic property in Persian medicine: a mechanistic review. *Journal of Thrombosis and Thrombolysis*.

[22] Choi S. H. (2008). WHO traditional medicine strategy and activities. "Standardization with evidence-based approaches". *Journal of Acupuncture and Meridian Studies*.

[23] Craig W. J. (1999). Health-promoting properties of common herbs. *American Journal of Clinical Nutrition*.

[24] Levine G. N., Bates E. R., Bittl J. A. (2016). 2016 ACC/AHA Guideline Focused Update on Duration of Dual Antiplatelet Therapy in Patients With Coronary Artery Disease: A Report of the American College of Cardiology/American Heart Association Task Force on Clinical Practice Guidelines: An Update of the 2011 ACCF/AHA/SCAI Guideline for Percutaneous Coronary Intervention, 2011 ACCF/AHA Guideline for Coronary Artery Bypass Graft Surgery, 2012 ACC/AHA/ACP/AATS/PCNA/SCAI/STS Guideline for the Diagnosis and Management of Patients With Stable Ischemic Heart Disease, 2013 ACCF/AHA Guideline for the Management of ST-Elevation Myocardial Infarction, 2014 AHA/ACC Guideline for the Management of Patients With Non-ST-Elevation Acute Coronary Syndromes, and 2014 ACC/AHA Guideline on Perioperative Cardiovascular Evaluation and Management of Patients Undergoing Noncardiac Surgery. *Circulation*.

[25] Levine G. N., Bates E. R., Blankenship J. C. (2016). 2015 ACC/AHA/SCAI focused update on primary percutaneous coronary intervention for patients with ST-elevation myocardial infarctionan: update of the 2011 ACCF/AHA/SCAI guideline for percutaneous coronary intervention and the 2013 ACCF/AHA guideline for the management of ST-elevation myocardial infarction: a report of the American college of cardiology/American Heart Association task force on clinical practice guidelines and the society for cardiovascular angiography and interventions. *Circulation*.

[26] Levine G. N., Bates E. R., Blankenship J. C. (2013). 2011 ACCF/AHA/SCAI guideline for percutaneous coronary intervention: a report of the American College of Cardiology Foundation/American Heart Association Task Force on Practice Guidelines and the Society for Cardiovascular Angiography and Interventions. *Catheterization and Cardiovascular Interventions*.

[27] The Task Force on Myocardial Revascularization of the European Society of Cardiology (ESC) (2010). Guidelines on myocardial revascularization. *European Journal of Cardio-Thoracic Surgery*.

[28] Jeon W. K., Lee J. H., Kim H. K. (2006). Anti-platelet effects of bioactive compounds isolated from the bark of *Rhus verniciflua* Stokes. *Journal of Ethnopharmacology*.

[29] Lee J.-C., Lim K.-T., Jang Y.-S. (2002). Identification of Rhus verniciflua Stokes compounds that exhibit free radical scavenging and anti-apoptotic properties. *Biochimica et Biophysica Acta (BBA) - General Subjects*.

[30] Adams J. D., Wang R., Yang J., Lien E. J. (2006). Preclinical and clinical examinations of Salvia miltiorrhiza and its tanshinones in ischemic conditions. *Chinese Medical Journal*.

[31] Zhou L., Zuo Z., Chow M. S. S. (2005). Danshen: an overview of its chemistry, pharmacology, pharmacokinetics, and clinical use. *Clinical Pharmacology and Therapeutics*.

[32] Albers G. W., Amarenco P., Easton J. D., Sacco R. L., Teal P. (2008). Antithrombotic and thrombolytic therapy for ischemic stroke: American College of Chest Physicians evidence-based clinical practice guidelines (8th edition). *Chest*.

[33] Lobo R., Prabhu K. S., Shirwaikar A. (2009). Curcuma zedoaria Rosc. (white turmeric): a review of its chemical, pharmacological and ethnomedicinal properties. *Journal of Pharmacy and Pharmacology*.

[34] Srivastava K. C., Bordia A., Verma S. K. (1995). Curcumin, a major component of food spice turmeric (*Curcuma longa*) inhibits aggregation and alters eicosanoid metabolism in human blood platelets. *Prostaglandins, Leukotrienes and Essential Fatty Acids*.

[35] Mayanglambam A., Dangelmaier C. A., Thomas D., Damodar Reddy C., Daniel J. L., Kunapuli S. P. (2010). Curcumin inhibits GPVI-mediated platelet activation by interfering with the kinase activity of Syk and the subsequent activation of PLC*γ*2. *Platelets*.

[36] Wang G.-S., Deng J.-H., Ma Y.-H., Shi M., Li B. (2012). Mechanisms, clinically curative effects, and antifungal activities of cinnamon oil and pogostemon oil complex against three species of Candida. *Journal of Traditional Chinese Medicine*.

[37] Chen S.-J., Wang M.-H., Chen I.-J. (1996). Antiplatelet and calcium inhibitory properties of eugenol and sodium eugenol acetate. *General Pharmacology: The Vascular System*.

[38] Raghavendra R. H., Naidu K. A. (2009). Spice active principles as the inhibitors of human platelet aggregation and thromboxane biosynthesis. *Prostaglandins, Leukotrienes and Essential Fatty Acids*.

[39] Parker S., May B., Zhang C., Zhang A. L., Lu C., Xue C. C. (2016). A Pharmacological Review of Bioactive Constituents of Paeonia lactiflora Pallas and Paeonia veitchii Lynch. *Phytotherapy Research*.

[40] Yasuda T., Kon R., Nakazawa T., Ohsawa K. (1999). Metabolism of paeonol in rats. *Journal of Natural Products*.

[41] Lee C. H., Kim J.-H. (2014). A review on the medicinal potentials of ginseng and ginsenosides on cardiovascular diseases. *Journal of Ginseng Research*.

[42] Lin H.-C., Ding H.-Y., Ko F.-N., Teng C.-M., Wu Y.-C. (1999). Aggregation inhibitory activity of minor acetophenones from Paeonia species. *Planta Medica*.

[43] Wang Y., Dan Y., Yang D. (2014). The genus Anemarrhena Bunge: a review on ethnopharmacology, phytochemistry and pharmacology. *Journal of Ethnopharmacology*.

[44] Son Y.-M., Jeong D.-H., Park H.-J., Rhee M.-H. (2017). The inhibitory activity of ginsenoside Rp4 in adenosine diphosphate-induced platelet aggregation. *Journal of Ginseng Research*.

[45] Zhou Q., Jiang L., Xu C. (2014). Ginsenoside Rg1 inhibits platelet activation and arterial thrombosis. *Thrombosis Research*.

[46] Iida Y., Oh K.-B., Saito M. (1999). Detection of antifungal activity in Anemarrhena asphodeloides by sensitive BCT method and isolation of its active compound. *Journal of Agricultural and Food Chemistry*.

[47] Kang L.-P., Zhang J., Cong Y. (2012). Steroidal glycosides from the rhizomes of anemarrhena asphodeloides and their antiplatelet aggregation activity. *Planta Medica*.

[48] Lu W.-Q., Qiu Y., Li T.-J., Tao X., Sun L.-N., Chen W.-S. (2011). Antiplatelet and antithrombotic activities of timosaponin B-II, an extract of Anemarrhena asphodeloides. *Clinical and Experimental Pharmacology and Physiology*.

[49] Muluye R. A., Bian Y., Alemu P. N. (2014). Anti-inflammatory and antimicrobial effects of heat-clearing Chinese herbs: a current review. *Journal of Traditional and Complementary Medicine*.

[50] Wang X., Zhang Y., Yang Y., Wu X., Fan H., Qiao Y. (2017). Identification of berberine as a direct thrombin inhibitor from traditional Chinese medicine through structural, functional and binding studies. *Scientific Reports*.

[51] Zhou X., Tang L., Xu Y., Zhou G., Wang Z. (2014). Towards a better understanding of medicinal uses of *Carthamus tinctorius* L. in traditional Chinese medicine: a phytochemical and pharmacological review. *Journal of Ethnopharmacology*.

[52] Li Y., Wang N. (2010). Antithrombotic effects of Danggui, Honghua and potential drug interaction with clopidogrel. *Journal of Ethnopharmacology*.

[53] Li H.-X., Han S.-Y., Wang X.-W. (2009). Effect of the carthamins yellow from *Carthamus tinctorius* L. on hemorheological disorders of blood stasis in rats. *Food and Chemical Toxicology*.

[54] Park H., Hwang Y., Ma J. Y. (2017). Single, repeated dose toxicity and genotoxicity assessment of herb formula KIOM2012H. *Integrative Medicine Research*.

[55] Patrono C., Rocca B. (2012). Aspirin and Other COX-1 Inhibitors. *Handb Exp Pharmacol*.

[56] Angiolillo D. J. (2012). The evolution of antiplatelet therapy in the treatment of acute coronary syndromes: From aspirin to the present day. *Drugs*.

[57] Warkentin T. E. (2012). Aspirin for dual prevention of venous and arterial thrombosis. *The New England Journal of Medicine*.

[58] Marcus A. J. (1977). Aspirin and Thromboembolism — A Possible Dilemma. *The New England Journal of Medicine*.

[59] Buczko W., Mogielnicki A., Kramkowski K., Chabielska E. (2003). Aspirin and the fibrinolytic response. *Thrombosis Research*.

[60] Kharbanda R. K., Walton B., Allen M. (2002). Prevention of inflammation-induced endothelial dysfunction: a novel vasculo-protective action of aspirin. *Circulation*.

[61] Antithrombotic Trialists' Collaboration (2002). Collaborative meta-analysis of randomised trials of antiplatelet therapy for prevention of death, myocardial infarction, and stroke in high risk patients. *British Medical Journal*.

[62] Eidelman R. S., Hebert P. R., Weisman S. M., Hennekens C. H. (2003). An update on aspirin in the primary prevention of cardiovascular disease. *JAMA Internal Medicine*.

[63] Landolfi R., Marchioli R., Kutti J. (2004). Efficacy and safety of low-dose aspirin in polycythemia vera. *The New England Journal of Medicine*.

[64] Derry S., Loke Y. K. (2000). Risk of gastrointestinal haemorrhage with long term use of aspirin: Meta-analysis. *British Medical Journal*.

[65] Cattaneo M. (2004). Aspirin and clopidogrel: efficacy, safety, and the issue of drug resistance. *Arteriosclerosis, Thrombosis, and Vascular Biology*.

[66] Savi P., Combalbert J., Gaich C. (1994). The antiaggregating activity of clopidogrel is due to a metabolic activation by the hepatic cytochrome P450-1A. *Thrombosis and Haemostasis*.

[67] Gur H., Wartenfeld R., Tanne D. (1998). Ticlopidine-induced severe neutropenia. *Postgraduate Medical Journal*.

[68] CAPRIE Steering Committee (1996). A randomised, blinded, trial of clopidogrel versus aspirin in patients at risk of ischaemic events (CAPRIE). *The Lancet*.

[69] Bennett C. L., Connors J. M., Carwile J. M. (2000). Thrombotic thrombocytopenic purpura associated with clopidogrel. *N Engl J Med*.

[70] Zakarija A., Bandarenko N., Pandey D. K. (2004). Clopidogrel-Associated TTP: An Update of Pharmacovigilance Efforts Conducted by Independent Researchers, Pharmaceutical Suppliers, and the Food and Drug Administration. *Stroke*.

[71] Angiolillo D. J., Capranzano P. (2008). Pharmacology of emerging novel platelet inhibitors. *American Heart Journal*.

[72] Jernberg T., Payne C. D., Winters K. J. (2006). Prasugrel achieves greater inhibition of platelet aggregation and a lower rate of non-responders compared with clopidogrel in aspirin-treated patients with stable coronary artery disease. *European Heart Journal*.

[73] Wiviott S. D., Trenk D., Frelinger A. L. (2007). Prasugrel compared with high loading- and maintenance-dose clopidogrel in patients with planned percutaneous coronary intervention: The prasugrel in comparison to clopidogrel for inhibition of platelet activation and aggregation-thrombolysis in myocardial infarction 44 trial. *Circulation*.

[74] Liverani E., Rico M. C., Garcia A. E., Kilpatrick L. E., Kunapuli S. P. (2013). Prasugrel metabolites inhibit neutrophil functions. *The Journal of Pharmacology and Experimental Therapeutics*.

[75] Wijeyeratne Y. D., Heptinstall S. (2011). Anti-platelet therapy: ADP receptor antagonists. *British Journal of Clinical Pharmacology*.

[76] Husted S., Van Giezen J. J. J. (2009). Ticagrelor: The first reversibly binding oral p2y12 receptor antagonist. *Cardiovascular Therapeutics*.

[77] van Giezen J. J. J., Nilsson L., Berntsson P. (2009). Ticagrelor binds to human P2Y12 independently from ADP but antagonizes ADP-induced receptor signaling and platelet aggregation. *Journal of Thrombosis and Haemostasis*.

[78] Capodanno D., Dharmashankar K., Angiolillo D. J. (2010). Mechanism of action and clinical development of ticagrelor, a novel platelet ADP P2Y12 receptor antagonist. *Expert Review of Cardiovascular Therapy*.

[79] Teng R., Butler K. (2010). Pharmacokinetics, pharmacodynamics, tolerability and safety of single ascending doses of ticagrelor, a reversibly binding oral P2Y12 receptor antagonist, in healthy subjects. *European Journal of Clinical Pharmacology*.

[80] Wallentin L., James S., Storey R. F. (2010). Effect of CYP2C19 and ABCB1 single nucleotide polymorphisms on outcomes of treatment with ticagrelor versus clopidogrel for acute coronary syndromes: A genetic substudy of the PLATO trial. *The Lancet*.

[81] Gurbel P. A., Bliden K. P., Butler K. (2009). Randomized double-blind assessment of the ONSET and OFFSET of the antiplatelet effects of ticagrelor versus clopidogrel in patients with stable coronary artery disease: The ONSET/OFFSET study. *Circulation*.

[82] Gurbel P. A., Bliden K. P., Butler K. (2010). Response to ticagrelor in clopidogrel nonresponders and responders and effect of switching therapies: the RESPOND study.. *Circulation*.

[83] Tantry U. S., Bliden K. P., Wei C. (2010). First analysis of the relation between CYP2C19 genotype and pharmacodynamics in patients treated with ticagrelor versus clopidogrel: The ONSET/OFFSET and RESPOND genotype studies. *Circulation: Cardiovascular Genetics*.

[84] Storey R. F., Angiolillo D. J., Patil S. B. (2010). Inhibitory effects of ticagrelor compared with clopidogrel on platelet function in patients with acute coronary syndromes: The PLATO (PLATelet inhibition and patient Outcomes) PLATELET substudy. *Journal of the American College of Cardiology*.

[85] Fuller R., Chavez B. (2012). Ticagrelor (brilinta), an antiplatelet drug for acute coronary syndrome. *P&T*.

[86] The EPIC Investigators (1994). Use of a Monoclonal Antibody Directed against the Platelet Glycoprotein IIb/IIIa Receptor in High-Risk Coronary Angioplasty. *The New England Journal of Medicine*.

[87] Bledzka K., Smyth S. S., Plow E. F. (2013). Integrin *α*IIb*β*3: from discovery to efficacious therapeutic target. *Circulation Research*.

[88] Braunwald E., Antman E. M., Beasley J. W. (2000). ACC/AHA guidelines for the management of patients with unstable angina and non-ST-segment elevation myocardial infarction. A report of the American College of Cardiology/American Heart Association Task Force on Practice Guidelines (Committee on the Management of Patients With Unstable Angina). *Journal of the American College of Cardiology*.

[89] Koutouzis M., Lagerqvist B., Oldgren J. (2010). Long-term results following switch from abciximab to eptifibatide during percutaneous coronary intervention. *Clinical Cardiology*.

[90] Topol E. J., Lincoff A. M., Kereiakes D. J. (2002). Multi-year follow-up of abciximab therapy in three randomized, placebo-controlled trials of percutaneous coronary revascularization. *The American Journal of Medicine*.

[91] Montalescot G. (2005). Three-year duration of benefit from abciximab in patients receiving stents for acute myocardial infarction in the randomized double-blind ADMIRAL study. *European Heart Journal*.

[92] Phillips D. R., Charo I. F., Scarborough R. M. (1991). GPIIb-IIIa: The responsive integrin. *Cell*.

[93] Scarborough R. M., Naughton M. A., Teng W. (1993). Design of potent and specific integrin antagonists. Peptide antagonists with high specificity for glycoprotein IIb-IIIa. *Journal of Biological Chemistry*.

[94] Lincoff A. M., Harrington R. A., Califf R. M. (2000). Management of patients with acute coronary syndromes in the United States by platelet glycoprotein IIb/IIIa inhibition. Insights from the platelet glycoprotein IIb/IIIa in unstable angina: receptor suppression using integrilin therapy (PURSUIT) trial. *Circulation*.

[95] Harrington R. A. (1997). Design and methodology of the PURSUIT trial: evaluating eptifibatide for acute ischemic coronary syndromes. Platelet Glycoprotein IIb-IIIa in Unstable Angina: Receptor Suppression Using Integrilin Therapy. *The American Journal of Cardiology*.

[96] Phillips D. R., Teng W., Arfsten A. (1997). Effect of Ca2+ on GP IIb-IIIa interactions with Integrilin: Enhanced GP IIb-IIIa binding and inhibition of platelet aggregation by reductions in the concentration of ionized calcium in plasma anticoagulated with citrate. *Circulation*.

[97] Ohman E. M., Kleiman N. S., Gacioch G. (1997). Combined accelerated tissue-plasminogen activator and platelet glycoprotein IIb/IIIa integrin receptor blockade with integrilin in acute myocardial infarction: results of a randomized, placebo-controlled, dose-ranging trial. *Circulation*.

[98] Harrington R. A., Kleiman N. S., Kottke-Marchant K. (1995). Immediate and reversible platelet inhibition after intravenous administration of a peptide glycoprotein IIb/IIIa inhibitor during percutaneous coronary intervention. *American Journal of Cardiology*.

[99] Tcheng J. E., Harrington R. A., Kottke-Marchant K. (1995). Multicenter, randomized, double-blind, placebo-controlled trial of the platelet integrin glycoprotein IIb/IIIa blocker Integrelin in elective coronary intervention. *Circulation*.

[100] Schulman S. P., Goldschmidt-Clermont P. J., Topol E. J. (1996). Effects of Integrelin, a platelet glycoprotein IIb/IIIa receptor antagonist, in unstable angina: A randomized multicenter trial. *Circulation*.

[101] Lynch Jr J. J., Cook J. J., Sitko G. R. (1995). Nonpeptide glycoprotein IIb/IIIa inhibitors. 5. Antithrombotic effects of MK-0383. *Journal of Pharmacology and Experimental Therapeutics*.

[102] Barrett J. S., Murphy G., Peerlinck K. (1994). Pharmacokinetics and pharmacodynamics of MK-383, a selective non-peptide platelet glycoprotein-IIb/IIIa receptor antagonist, in healthy men. *Clinical Pharmacology & Therapeutics*.

[103] Vickers S., Theoharides A. D., Arison B. (1999). In vitro and in vivo studies on the metabolism of tirofiban. *Drug Metabolism and Disposition*.

[104] Schneider D. J. (2011). Anti-platelet therapy: Glycoprotein IIb-IIIa antagonists. *British Journal of Clinical Pharmacology*.

[105] Kim J. H., Shin Y. C., Ko S.-G. (2014). Integrating traditional medicine into modern inflammatory diseases care: multitargeting by rhus verniciflua stokes. *Mediators of Inflammation*.

[106] Aggarwal B. B., Ichikawa H., Garodia P. (2006). From traditional Ayurvedic medicine to modern medicine: Identification of therapeutic targets for suppression of inflammation and cancer. *Expert Opinion on Therapeutic Targets*.

[107] McEwen B. J. (2015). The influence of herbal medicine on platelet function and coagulation: a narrative review. *Seminars in Thrombosis and Hemostasis*.

[108] Duke J. A. (2002). *Handbook of Medicinal Herbs*.

[109] Kim J. H., Go H. Y., Jin D. H. (2008). Inhibition of the PI3K-Akt/PKB survival pathway enhanced an ethanol extract of *Rhus verniciflua* Stokes-induced apoptosis via a mitochondrial pathway in AGS gastric cancer cell lines. *Cancer Letters*.

[110] He J.-B., Luo J., Zhang L., Yan Y.-M., Cheng Y.-X. (2013). Sesquiterpenoids with new carbon skeletons from the resin of Toxicodendron vernicifluum as new types of extracellular matrix inhibitors. *Organic Letters*.

[111] Lee J.-C., Lee K.-Y., Kim J. (2004). Extract from Rhus verniciflua Stokes is capable of inhibiting the growth of human lymphoma cells. *Food and Chemical Toxicology*.

[112] Kim I. T., Park Y. M., Shin K. M. (2004). Anti-inflammatory and anti-nociceptive effects of the extract from Kalopanax pictus, Pueraria thunbergiana and Rhus verniciflua. *Journal of Ethnopharmacology*.

[113] Jeong S.-J., Park J.-G., Kim S. (2015). Extract of Rhus verniciflua stokes protects the diet-induced hyperlipidemia in mice. *Archives of Pharmacal Research*.

[114] Lee J., Kim J., Jang Y. (2003). Ethanol-eluted Extract of Rhus verniciflua Stokes Inhibits Cell Growth and Induces Apoptosis in Human Lymphoma Cells. *BMB Reports*.

[115] Lim K. T., Hu C., Kitts D. D. (2001). Antioxidant activity of a *Rhus verniciflua* Stokes ethanol extract. *Food and Chemical Toxicology*.

[116] Xie Y., Zhang J., Liu W., Xie N., Feng F., Qu W. (2016). New urushiols with platelet aggregation inhibitory activities from resin of Toxicodendron vernicifluum. *Fitoterapia*.

[117] Son Y. O., Lee K. Y., Lee J. C. (2005). Selective antiproliferative and apoptotic effects of flavonoids purified from Rhus verniciflua Stokes on normal versus transformed hepatic cell lines. *Toxicology Letters*.

[118] Chen X., Guo J., Bao J., Lu J., Wang Y. (2014). The anticancer properties of Salvia miltiorrhiza Bunge (Danshen): a systematic review. *Medicinal Research Reviews*.

[119] Chan T. Y. K. (2001). Interaction between warfarin and danshen (*Salvia miltiorrhiza*). *Annals of Pharmacotherapy*.

[120] Liu J., Shen H.-M., Ong C.-N. (2000). *Salvia miltiorrhiza* inhibits cell growth and induces apoptosis in human hepatoma HepG2 cells. *Cancer Letters*.

[121] Ji X. Y., Tan B. K., Zhu Y. Z. (2000). Salvia miltiorrhiza and ischemic diseases. *Acta Pharmacologica Sinica*.

[122] Song Y.-H., Liu Q., Lv Z.-P., Chen Y.-Y., Zhou Y.-C., Sun X.-G. (2008). Protection of a polysaccharide from Salvia miltiorrhiza, a Chinese medicinal herb, against immunological liver injury in mice. *International Journal of Biological Macromolecules*.

[123] Chen W., Chen G. (2017). Danshen (Salvia miltiorrhiza bunge): A prospective healing sage for cardiovascular diseases. *Current Pharmaceutical Design*.

[124] Park J.-W., Lee S.-H., Yang M.-K. (2008). 15,16-Dihydrotanshinone I, a major component from Salvia miltiorrhiza Bunge (Dansham), inhibits rabbit platelet aggregation by suppressing intracellular calcium mobilization. *Archives of Pharmacal Research*.

[125] Cheng T. O. (2007). Cardiovascular effects of Danshen. *International Journal of Cardiology*.

[126] Toegel S., Wu S. Q., Otero M. (2012). Caesalpinia sappan extract inhibits IL1*β*-mediated overexpression of matrix metalloproteinases in human chondrocytes. *Genes & Nutrition*.

[127] Lee M.-J., Lee H.-S., Jung H.-J. (2010). Caesalpinia sappan L. ameliorates hypercholesterolemia in C57BL/6 mice and suppresses inflammatory responses in human umbilical vein endothelial cells (HUVECs) by antioxidant mechanism. *Immunopharmacology and Immunotoxicology*.

[128] Lee M. J., Lee H. S., Kim H. (2010). Antioxidant properties of benzylchroman derivatives from Caesalpinia sappan L. against oxidative stress evaluated in vitro. *Journal of Enzyme Inhibition and Medicinal Chemistry*.

[129] Moon H. I., Chung I. M., Seo S. H., Kang E. Y. (2010). Protective effects of 3′-deoxy-4-O-methylepisappanol from Caesalpinia sappan against glutamate-induced neurotoxicity in primary cultured rat cortical cells. *Phytotherapy Research*.

[130] Wang Y.-Z., Sun S.-Q., Zhou Y.-B. (2011). Extract of the dried heartwood of Caesalpinia sappan L. attenuates collagen-induced arthritis. *Journal of Ethnopharmacology*.

[131] Kim S.-H., Kim B., Kim S.-H. (2012). Brazilin induces apoptosis and G2/M arrest via inactivation of histone deacetylase in multiple myeloma U266 cells. *Journal of Agricultural and Food Chemistry*.

[132] Lee C.-C., Wang C.-N., Kang J.-J. (2012). Antiallergic asthma properties of Brazilin through inhibition of TH2 responses in T cells and in a murine model of asthma. *Journal of Agricultural and Food Chemistry*.

[133] Xu H.-X., Lee S. F. (2004). The antibacterial principle of Caesalpina sappan. *Phytotherapy Research*.

[134] You E.-J., Khil L.-Y., Kwak W.-J. (2005). Effects of brazilin on the production of fructose-2,6-bisphosphate in rat hepatocytes. *Journal of Ethnopharmacology*.

[135] Hwang G.-S., Kim J.-Y., Chang T.-S., Jeon S.-D., So D.-S., Moon C.-K. (1998). Effects of brazilin on the phospholipase A2 activity and changes of intracellular free calcium concentration in rat platelets. *Archives of Pharmacal Research*.

[136] Itokawa H., Shi Q., Akiyama T., Morris-Natschke S. L., Lee K.-H. (2008). Recent advances in the investigation of curcuminoids. *Chinese Medicine*.

[137] Khar A., Ali A. M., Pardhasaradhi B. V. V., Begum Z., Anjum R. (1999). Antitumor activity of curcumin is mediated through the induction of apoptosis in AK-5 tumor cells. *FEBS Letters*.

[138] Ozaki Y. (1990). Antiinflammatory effect of Curcuma xanthorrhiza Roxb, and its active principles. *Chemical & Pharmaceutical Bulletin*.

[139] Kasahara K., Nomura S., Subeki (2005). Anti-babesial compounds from Curcuma zedoaria. *Planta Medica*.

[140] Syu W.-J., Shen C.-C., Don M.-J., Ou J.-C., Lee G.-H., Sun C.-M. (1998). Cytotoxicity of curcuminoids and some novel compounds from Curcuma zedoaria. *Journal of Natural Products*.

[141] Gonda R., Tomoda M., Ōhara N., Takada K. (1993). Arabinogalactan Core Structure and Immunological Activities of Ukonan C, an Acidic Polysaccharide from the Rhizome of Curcuma longa. *Biological & Pharmaceutical Bulletin*.

[142] Chen Z., Ao J., Yang W., Jiao L., Zheng T., Chen X. (2013). Purification and characterization of a novel antifungal protein secreted by Penicillium chrysogenum from an Arctic sediment. *Applied Microbiology and Biotechnology*.

[143] Keihanian F., Saeidinia A., Bagheri R. K., Johnston T. P., Sahebkar A. (2018). Curcumin, hemostasis, thrombosis, and coagulation. *Journal of Cellular Physiology*.

[144] Ghandadi M., Sahebkar A. (2017). Curcumin: An effective inhibitor of interleukin-6. *Current Pharmaceutical Design*.

[145] Panahi Y., Khalili N., Sahebi E. (2017). Antioxidant effects of curcuminoids in patients with type 2 diabetes mellitus: a randomized controlled trial. *Inflammopharmacology*.

[146] Menon V. P., Sudheer A. R. (2007). Antioxidant and anti-inflammatory properties of curcumin. *Advances in Experimental Medicine and Biology*.

[147] Cheng A. L., Hsu C. H., Lin J. K. (2001). Phase I clinical trial of curcumin, a chemopreventive agent, in patients with high-risk or pre-malignant lesions. *Anticancer Research*.

[148] Shah B. H., Nawaz Z., Pertani S. A. (1999). Inhibitory effect of curcumin, a food spice from turmeric, on platelet-activating factor- and arachidonic acid-mediated platelet aggregation through inhibition of thromboxane formation and Ca^2+^ signaling. *Biochemical Pharmacology*.

[149] Ho S.-C., Chang K.-S., Chang P.-W. (2013). Inhibition of neuroinflammation by cinnamon and its main components. *Food Chemistry*.

[150] Akilen R., Tsiami A., Devendra D., Robinson N. (2010). Glycated haemoglobin and blood pressure-lowering effect of cinnamon in multi-ethnic Type 2 diabetic patients in the UK: A randomized, placebo-controlled, double-blind clinical trial. *Diabetic Medicine*.

[151] Ziment I. (1991). History of the treatment of chronic bronchitis. *Respiration*.

[152] Nakano K., Nohara T., Tomimatsu T., Nishioka I. (1981). Studies on the constituents of Cinnamomi Cortex. VI. On the mass spectrometry of Cassia diterpene (lactone type). *Yakugaku Zasshi*.

[153] Tran M. N., Lee I., Do T. H., Kim H., Min B., Bae K. (2009). Tyrosinase-inhibitory constituents from the twigs of Cinnamomum cassia. *Journal of Natural Products*.

[154] Yeh H.-F., Luo C.-Y., Lin C.-Y., Cheng S.-S., Hsu Y.-R., Chang S.-T. (2013). Methods for thermal stability enhancement of leaf essential oils and their main constituents from indigenous cinnamon (Cinnamomum osmophloeum). *Journal of Agricultural and Food Chemistry*.

[155] Singh H. B., Srivastava M., Singh A. B., Srivastava A. K. (1995). Cinnamon bark oil, a potent fungitoxicant against fungi causing respiratory tract mycoses. *Allergy*.

[156] Lin C.-C., Wu S.-J., Chang C.-H., Ng L.-T. (2003). Antioxidant activity of Cinnamomum cassia. *Phytotherapy Research*.

[157] Lee S., Han J.-M., Kim H. (2004). Synthesis of cinnamic acid derivatives and their inhibitory effects on LDL-oxidation, acyl-CoA:cholesterol acyltransferase-1 and -2 activity, and decrease of HDL-particle size. *Bioorganic & Medicinal Chemistry Letters*.

[158] Cheng S.-S., Liu J.-Y., Tsai K.-H., Chen W.-J., Chang S.-T. (2004). Chemical composition and mosquito larvicidal activity of essential oils from leaves of different Cinnamomum osmophloeum provenances. *Journal of Agricultural and Food Chemistry*.

[159] Kim S. Y., Koo Y. K., Koo J. Y. (2010). Platelet anti-aggregation activities of compounds from Cinnamomum cassia. *Journal of Medicinal Food*.

[160] Koo Y. K., Kim J. M., Koo J. Y. (2010). Platelet anti-aggregatory and blood anti-coagulant effects of compounds isolated from Paeonia lactiflora and Paeonia suffruticosa. *Pharmazie*.

[161] Chou T.-C. (2003). Anti-inflammatory and analgesic effects of paeonol in carrageenan-evoked thermal hyperalgesia. *British Journal of Pharmacology*.

[162] Ke Z., Wang G., Yang L. (2017). Crude terpene glycoside component from Radix paeoniae rubra protects against isoproterenol-induced myocardial ischemic injury via activation of the PI3K/AKT/mTOR signaling pathway. *Journal of Ethnopharmacology*.

[163] Xie P., Cui L., Shan Y., Kang W.-Y. (2017). Antithrombotic effect and mechanism of radix paeoniae rubra. *BioMed Research International*.

[164] Hirai A., Terano T., Hamazaki T. (1983). Studies on the mechanism of antiaggregatory effect of moutan cortex. *Thrombosis Research*.

[165] Li X.-T., Chen R., Jin L.-M., Chen H.-Y. (2009). Regulation on energy metabolism and protection on mitochondria of Panax Ginseng polysaccharide. *American Journal of Chinese Medicine*.

[166] Samukawa K., Suzuki Y., Ohkubo N., Aoto M., Sakanaka M., Mitsuda N. (2008). Protective effect of ginsenosides Rg2 and Rh1 on oxidation-induced impairment of erythrocyte membrane properties. *Biorheology*.

[167] Zhang Y.-G., Zhang H.-G., Zhang G.-Y. (2008). Panax notoginseng saponins attenuate atherosclerosis in rats by regulating the blood lipid profile and an anti-inflammatory action. *Clinical and Experimental Pharmacology and Physiology*.

[168] Lau A.-J., Toh D.-F., Chua T.-K., Pang Y.-K., Woo S.-O., Koh H.-L. (2009). Antiplatelet and anticoagulant effects of Panax notoginseng: Comparison of raw and steamed Panax notoginseng with Panax ginseng and Panax quinquefolium. *Journal of Ethnopharmacology*.

[169] Jin Y. R., Yu J. Y., Lee J. J. (2007). Antithrombotic and antiplatelet activities of Korean red ginseng extract. *Basic & Clinical Pharmacology & Toxicology*.

[170] Lee S. M., Bae B.-S., Park H.-W. (2015). Characterization of Korean red ginseng (*Panax ginseng* Meyer): history, preparation method, and chemical composition. *Journal of Ginseng Research*.

[171] Jeong D., Irfan M., Kim S.-D. (2017). Ginsenoside Rg3-enriched red ginseng extract inhibits platelet activation and in vivo thrombus formation. *Journal of Ginseng Research*.

[172] Takahashi M., Konno C., Hikino H. (1985). Isolation and hypoglycemic activity of anemarans A, B, C and D, glycans of Anemarrhena asphodeloides rhizomes. *Planta Medica*.

[173] Niwa A., Takeda O., Ishimaru M. (1988). Screening test for platelet aggregation inhibitor in natural products. The active principle of Anemarrhenae Rhizoma. *Yakugaku Zasshi*.

[174] Bhattacharya S. K., Ghosal S., Chaudhuri R. K., Sanyal A. K. (1972). Canscora decussata (Gentianaceae) xanthones. III: Pharmacological studies. *Journal of Pharmaceutical Sciences*.

[175] Takeda Y., Togashi H., Matsuo T., Shinzawa H., Takeda Y., Takahashi T. (2001). Growth inhibition and apoptosis of gastric cancer cell lines by Anemarrhena asphodeloides Bunge. *Journal of Gastroenterology*.

[176] Dong J., Han G. Y. (1991). A new active steroidal saponin from Anemarrhena asphodeloides. *Planta Medica*.

[177] Zhang J., Meng Z., Zhang M., Ma D., Xu S., Kodama H. (1999). Effect of six steroidal saponins isolated from anemarrhenae rhizoma on platelet aggregation and hemolysis in human blood. *Clinica Chimica Acta*.

[178] Ikram M. (1975). A review on the chemical and pharmacological aspects of genus Berberis. *Planta Medica*.

[179] Schmeller T., Latz-Brüning B., Wink M. (1997). Biochemical activities of berberine, palmatine and sanguinarine mediating chemical defence against microorganisms and herbivores. *Phytochemistry*.

[180] Kettmann V., Kosfalova D., Jantova S., Cernakova M., Drimal J. (2004). In vitro cytotoxicity of berberine against HeLa and L1210 cancer cell lines. *Pharmazie*.

[181] Kuo C.-L., Chi C.-W., Liu T.-Y. (2004). The anti-inflammatory potential of berberine in vitro and in vivo. *Cancer Letters*.

[182] Su Y., Wang Q., Wang C., Chan K., Sun Y., Kuang H. (2014). The treatment of Alzheimer's disease using Chinese medicinal plants: from disease models to potential clinical applications. *Journal of Ethnopharmacology*.

[183] Kong W., Wei J., Abidi P. (2004). Berberine is a novel cholesterol-lowering drug working through a unique mechanism distinct from statins. *Nature Medicine*.

[184] Zhou L., Yang Y., Wang X. (2007). Berberine stimulates glucose transport through a mechanism distinct from insulin. *Metabolism*.

[185] Wang Y., Huang Y., Lam K. S. L. (2009). Berberine prevents hyperglycemia-induced endothelial injury and enhances vasodilatation via adenosine monophosphate-activated protein kinase and endothelial nitric oxide synthase. *Cardiovascular Research*.

[186] Zeng X.-H., Zeng X.-J., Li Y.-Y. (2003). Efficacy and safety of berberine for congestive heart failure secondary to ischemic or idiopathic dilated cardiomyopathy. *American Journal of Cardiology*.

[187] Affuso F., Mercurio V., Fazio V., Fazio S. (2010). Cardiovascular and metabolic effects of Berberine. *World Journal of Cardiology*.

[188] Huang C. G., Chu Z. L., Yang Z. M. (1991). Effects of berberine on synthesis of platelet TXA2 and plasma PGI2 in rabbits. *Zhongguo Yao Li Xue Bao*.

[189] Asgarpanah J., Kazemivash N. (2013). Phytochemistry, pharmacology and medicinal properties of *Carthamus tinctorius* L.. *Chinese Journal of Integrative Medicine*.

[190] Kim H. J., Bae Y. C., Park R. W. (2002). Bone-protecting effect of safflower seeds in ovariectomized rats. *Calcified Tissue International*.

[191] Yuk T. H., Kang J. H., Lee S. R. (2002). Inhibitory effect of Carthamus tinctorius L. seed extracts on bone resorption mediated by tyrosine kinase, COX-2 (cyclooxygenase) and PG (prostaglandin) E2. *American Journal of Chinese Medicine*.

[192] Hong B., Wang Z., Xu T., Li C., Li W. (2015). Matrix solid-phase dispersion extraction followed by high performance liquid chromatography-diode array detection and ultra performance liquid chromatography-quadrupole-time of flight-mass spectrometer method for the determination of the main compounds from Carthamus tinctorius L. (Hong-hua). *Journal of Pharmaceutical and Biomedical Analysis*.

[193] Qu C., Wang L.-Y., Lin H. (2017). Hierarchical identification of bioactive components in a medicinal herb by preparative high-performance liquid chromatography and selective knock-out strategy. *Journal of Pharmaceutical and Biomedical Analysis*.

[194] Choi D. W., Kim J. H., Cho S. Y., Kim D. H., Chang S. Y. (2002). Regulation and quality control of herbal drugs in Korea. *Toxicology*.

